# A novel capsid-modified oncolytic recombinant adenovirus type 5 for tumor-targeting gene therapy by intravenous route

**DOI:** 10.18632/oncotarget.10075

**Published:** 2016-06-15

**Authors:** Zhen Wang, Bin Yu, Baoming Wang, Jingyi Yan, Xiao Feng, Zixuan Wang, Lizheng Wang, Haihong Zhang, Hui Wu, Jiaxin Wu, Wei Kong, Xianghui Yu

**Affiliations:** ^1^ National Engineering Laboratory for AIDS Vaccine, School of Life Sciences, Jilin University, Changchun, 130012, China; ^2^ Key Laboratory for Molecular Enzymology and Engineering, The Ministry of Education, Jilin University, Changchun, 130012, China

**Keywords:** oncolytic adenovirus, gene therapy, capsid protein IX, TRAIL

## Abstract

Oncolytic adenovirus (Ad)-vectored gene therapy is a promising strategy for cancer treatment. However, the lack of cancer cell selectivity or tumor tissue specificity of Ads limits their clinical application by intravenous (IV) injection. In this paper, a novel recombinant Ad5 vector was constructed carrying the capsid protein IX modified by the tumor necrosis factor related apoptosis-inducing ligand (TRAIL), which targets tumor cells bearing high levels of its receptor far above those of normal cells. Specific association of the Ad virion with TRAIL was achieved using synthetic leucine zipper-like dimerization domains (zippers). Analysis of the chemical properties of the modified recombinant Ad (rAd5pz-zTRAIL-RFP) showed that the TRAIL protein was present on the surface of purified virus particles, and it could induce apoptosis of infected cancer cells prior to expression of foreign genes. We also constructed a novel modified recombinant oncolytic Ad (rAd5pz-zTRAIL-RFP-SΔ24E1a) which showed significantly enhanced anti-tumor effects both *in vitro* and *in vivo* by linkage of TRAIL to the viral capsid. Moreover, rAd5pz-zTRAIL-RFP-SΔ24E1a showed significantly improved tumor tissue targeting and reduced liver tropism when IV injected *in vivo*. Thus, we successfully obtained new oncolytic Ad5 gene therapy vectors with enhanced targeting and efficacy, providing a platform for further clinical application of Ad vectors for cancer treatment.

## INTRODUCTION

Viruses are widely used to develop replicative vector therapies for cancer (virotherapy and oncolysis) [1, 2]. Oncolytic adenoviruses (Ads), also known as conditionally replicating Ads (CRAds), are among the most popular viruses used for cancer gene therapy [3–5]. Their main advantage is selective viral replication in tumor cells but not in normal cells. Therefore, oncolytic Ads have been designed to specifically kill tumor cells as a direct consequence of viral infection [6]. The anti-tumor activity of an oncolytic Ad relies on efficient and specific delivery of the vector to cancer cells or tissues for safe and potent therapeutic effects [7].

To achieve an effective Ad-based cancer therapy, systemic (i.e., intravenous, IV) administration is the ideal mode of delivery [8]. Many experiments have been conducted using Ad vectors to treat liver cancer, some of which resulted in the successful removal of liver tumor cells [7, 9]. A practical explanation for this result is the retention of greater than 80% of the systemically injected Ad in the liver [10], because the binding of Ad5 with FX through FX Gla domain and the Hexon HVRs can deliver adenovirus to hepatocytes existing in the bloodstream *in vivo* [11–13]. However, such liver tropism poses a problem when using Ad vectors targeting tumors in other tissues. Current efforts encourage the use of Ad capsid modifications with translational research tools to address the ample challenges in this field. Since pIX is exposed on the surface of the virion, its mutants have been used as a platform for ligand insertion at its C terminus, with the aim of developing cell-targeted vectors for gene therapy [14, 15].

Over the past 10 years, tumor necrosis factor-related apoptosis-inducing ligand (TRAIL) has emerged as a promising candidate for cancer therapy based on inducing apoptosis specifically in various tumor cells without significant toxicity toward normal cells [16, 17]. TRAIL induces an extrinsic apoptotic signal in cancer cells due to the higher frequency of death receptors (DR4, DR5) expressed on their surface compared with normal cells [18–21]. And some studies indicated that TRAIL could target to death receptors on tumor cell surface [22]. However, TRAIL also has obvious drawbacks, namely a short *in vivo* half-life and low specific bioactivity [23–25]. We hypothesized that a tumor-targeted Ad vector can be achieved via highly specific association with secreted bioactive TRAIL proteins by employing synthetic leucine zipper-like dimerization domains (zippers) that have been optimized for structural compatibility between the Ad capsid and TRAIL. The feasibility and effectiveness of such strategy has been confirmed by M. N. Garas' study recently [26]. In this report, based on a 24-base-pair deletion mutant E1A oncolytic Ad (Δ24E1A) [27–29], we outlined the biochemical analysis, functional validation and anti-tumor activity of a novel TRAIL-modified Ad vector and demonstrated that this engineered Ad virion with TRAIL on the surface could target cancer tissues administered by IV injection *in vivo*.

## RESULTS

### Construction of an Ad vector coupled with TRAIL using leucine zipper heterodimers

**Figure 1:**
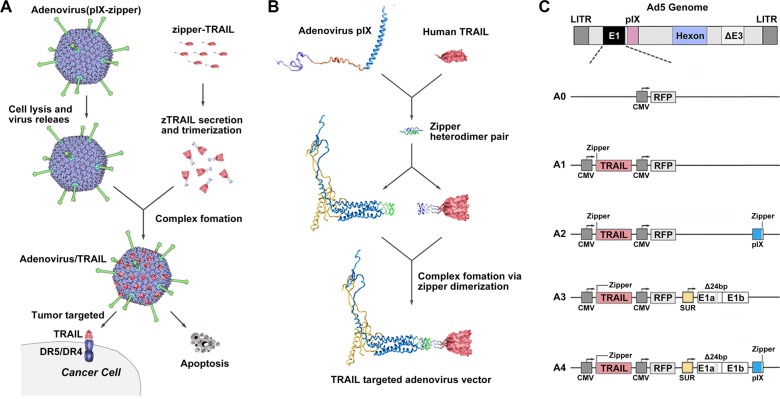
Diagrams of pIX-zipper-TRAIL configuration and Ad vector genomes depicting tumor targeting strategy (**A**) Schematic of proposed Ad vector targeting approach showing Ad-mediated TRAIL expression and secretion, followed by cell lysis, Ad virion release and extracellular formation of a targeted Ad vector/TRAIL complex. (**B**) Configuration of heterodimeric zipper domains, in which one zipper domain was genetically incorporated into the C-terminus of pIX, and its counterpart was fused to the N-terminus of a recombinant TRAIL molecule. (**C**) Schematic diagram of recombinant Ads depicting the organization of elements. A0, rAd5-RFP; A1, rAd5-zTRAIL-RFP; A2, rAd5pz-zTRAIL-RFP; A3, rAd5-zTRAIL-RFP-Δ24E1a; A4, rAd5pz-zTRAIL-RFP-Δ24E1a.

The TRAIL trimer has demonstrated potent anti-tumor activity in cancer models based on tumor cell lines and primary tumor samples. Interestingly, at the C termini of four pIX monomers, three of them are oriented in the same direction [30]. We expected that the expression and secretion of TRAIL would be followed by cell lysis, Ad virion release and then coupling of TRAIL with pIX to form an anti-tumor Ad vector/TRAIL complex (Figure 1A). Towards this goal, we used two heterodimeric zipper pairs (E·E34/R·R34) [31], wherein one zipper domain was genetically incorporated onto the C-terminus of the pIX, and its counterpart was fused to the N-terminus of TRAIL (Figure 1B). The CMV-zTRAIL expression cassette was inserted in the deleted E1 region, and CMV-RFP was used as a reporter gene (Figure 1C). The resulting recombinant Ad was designated rAd5pz-zTRAIL-RFP, while rAd5-RFP and rAd5-zTRAIL-RFP were constructed at the same time as controls.

### Biochemical analysis and functional validation of TRAIL on surface of recombinant Ad particles

**Figure 2:**
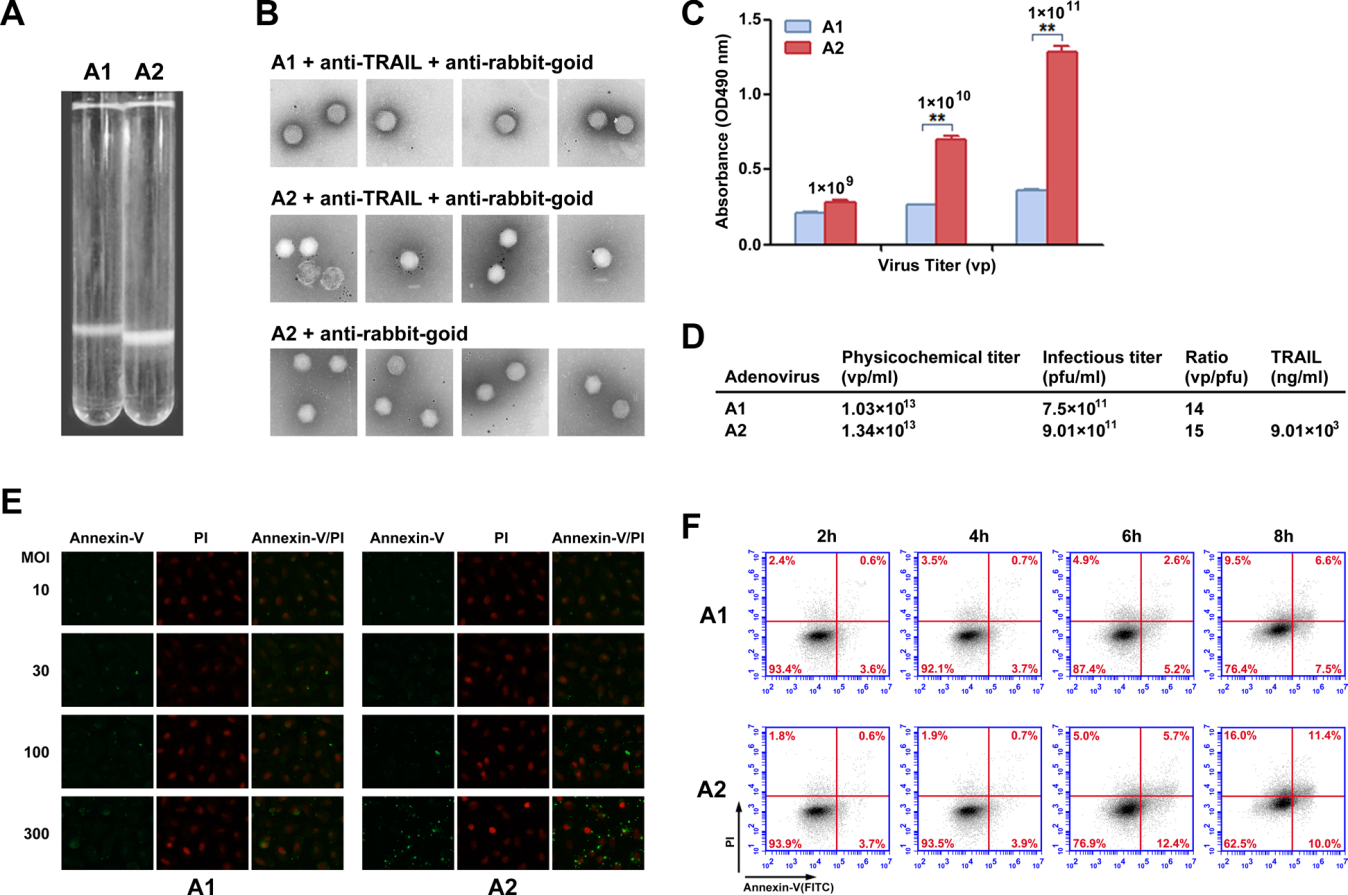
Chemical property analysis and biological activity of recombinant Ad vectors (**A**) rAd5-zTRAIL-RFP (**A1**) and rAd5pz-zTRAIL-RFP (**A2**) purified by a second round of CsCl continuous density gradient centrifugation. The Ads are located in the white bands. (**B**) Immunogold labeling of rAd5-zTRAIL-RFP and rAd5pz-zTRAIL-RFP viruses reacted with anti-rabbit gold-coupled IgG with or without primary anti-TRAIL antibody. The rAd5-zTRAIL-RFP virus was incubated with the anti-TRAIL antibody and anti-rabbit IgG antibody coupled to 6-nm gold particles (top row), rAd5pz-zTRAIL-RFP was incubated with both primary and secondary antibodies (bottom row). A rAd5pz-zTRAIL-RFP control sample was reacted only with the secondary antibody (middle row). (**C**) The content of TRAIL on the modified virus surface was detected by ELISA. **P < 0.01. (**D**) The physicochemical properties of purified virus include physical titers and infectious titers, and ratios among them, and TRAIL content. (**E**) Apoptosis of ZR-75-30 cells induced by different MOIs and times were determined by fluorescence microscopy using the Annexin-V/PI reagent kit. Fluorescence images at 400× magnification showed changes of recombinant Ad-infected tumor cells stained with Annexin-V/PI. (**F**) ZR-75-30 cells treated with two viruses (rAd5-zTRAIL-RFP, rAd5pz-zTRAIL-RFP) at 100 MOI for 2, 4, 6 and 8 h. The cells were analyzed using the apoptosis assay kit and flow cytometry.

Recombinant viruses were produced and analyzed to confirm the presence of TRAIL on the viral particle surface. If TRAIL was effectively linked to the viral capsid, the Ad particle size was expected to change. In the second purification by continuous CsCl density gradient centrifugation, the modified recombinant Ad (rAd5pz-zTRAIL-RFP) settled lower in the gradient compared to the control (rAd5-zTRAIL-RFP) (Figure 2A), indicating an increase in viral particle (VP) size. In order to further confirm that on the virions surface could display pIX-TRAIL, immunogold electron microscopy was performed to directly visualize rAd5s (Figure 2B). Anti-TRAIL antibody, followed by a secondary antibody conjugated with 5 nm gold particles, was used to detect pIX-TRAIL. No antibody binding was detected with the rAd5-zTRAIL-RFP. rAd5pz-zTRAIL-RFP was recognized by the corresponding primary and secondary antibodies, and it was not recognized by only the secondary antibody. And we also found that the absorbance was higher and increase with the quantity of rAd5pz-zTRAIL-RFP, compared with rAd5-zTRAIL-RFP in ELISA (Figure 2C). These results showed that TRAIL was present on the viral particles with modified pIX. Then we analyzed the titers of physics and infection, and results showed that the yields of rAd5pz-zTRAIL-RFP were not affected by modified TRAIL compared with rAd5-zTRAIL-RFP (Figure 2D). A sandwich ELISA was used to quantitatively analyze the content of TRAIL on the modified virus surface. And almost 40 copies of TRAIL proteins per virus particle were found, since the TRAIL concentration was 9.01 × 10^3^ ng/ml while the titer of rAd5pz-zTRAIL-RFP was 1.34 × 10^13^ vp/ml (Figure 2D).

To further examine the effect of TRAIL on the viral surface in infected cells, we compared the abilities of rAd5-zTRAIL-RFP and rAd5pz-zTRAIL-RFP to induce apoptosis at the time of the initial virus infection when exogenous TRAIL gene expression had not started. We first employed a fluorescence assay to evaluate rAd5-zTRAIL-RFP and rAd5pz-zTRAIL-RFP capacity to induce apoptosis of tumor cells. Recombinant Ads were used to infect tumor cells at various infectious doses for 6 h. As evidenced by microscopic observation of green fluorescence cells at 6 h, rAd5pz-zTRAIL-RFP could not induce apoptosis in ZR-75-30 cells at the low MOI of 10 or 30, and numerous apoptosis cells appeared at MOI of 100 and 300 (Figure 2E). To evaluate the utility of rAd5pz-zTRAIL-RFP and further explore the role of TRAIL on the virus surface over time, the apoptosis rate of infected cells was measured by flow cytometry. The presence of TRAIL proved to be instrumental in the induction of apoptosis, as 18.1% of ZR-75-30 cells cultured with rAd5pz-zTRAIL-RFP were stained with Annexin-V after 6 h of incubation (Figure 2F). After 6 h, cells infected with rAd5pz-zTRAIL-RFP, but not with rAd5-zTRAIL-RFP, showed an increasing trend towards the late apoptosis stage (upper right quadrant on the plot) (Figure 2F). The results also showed that TRAIL coupling to virus particles by fusing with zipper still maintained its biological activities.

### Analysis of replication, expression and cytotoxicity of modified oncolytic Ad *in vitro*

**Figure 3:**
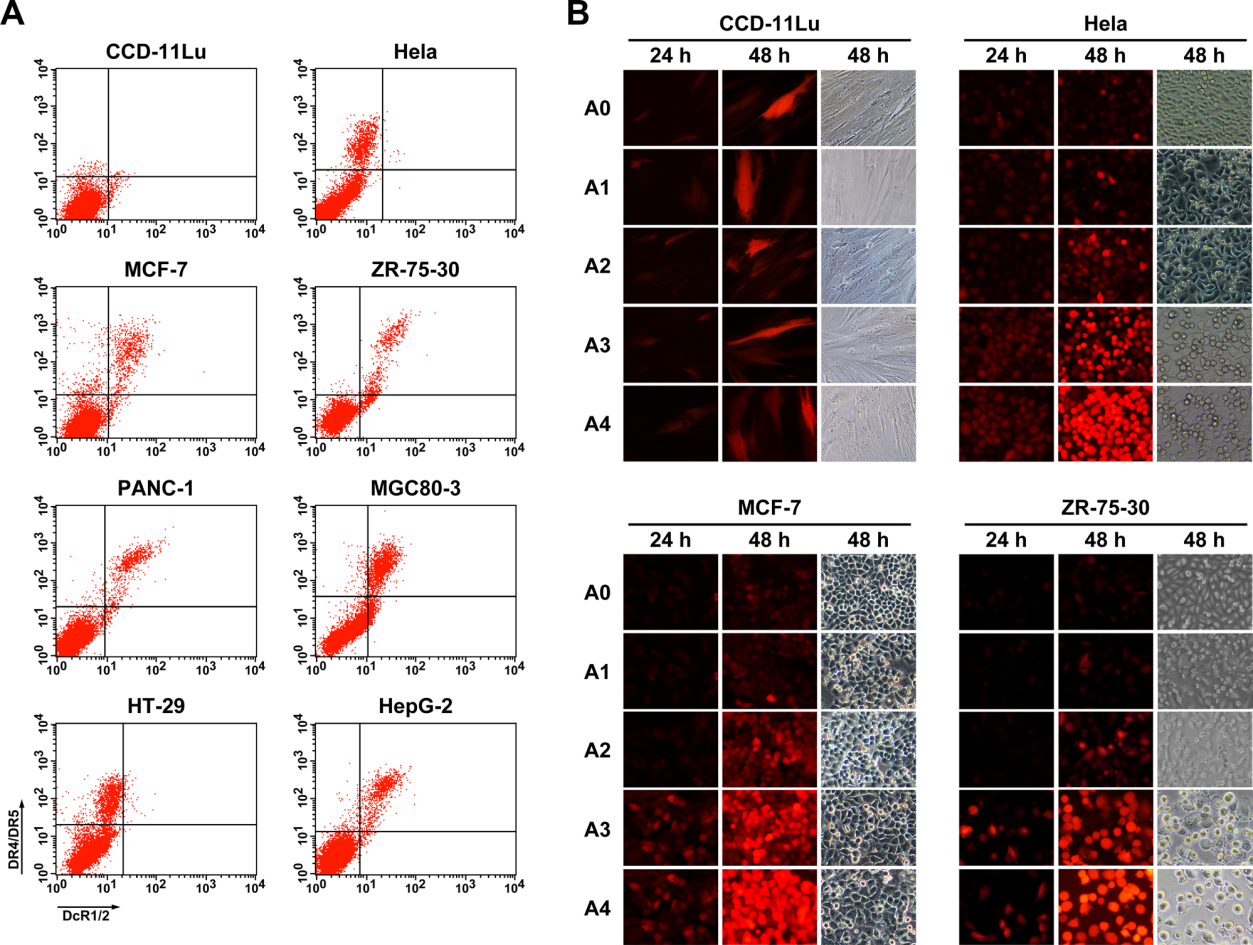
Expression of TRAIL receptors on normal cells and cancer cells, and *in vitro* detection of fluorescently labeled proteins in infected cells (**A**) Cells were labeled with goat anti-DcR1/2 polyclonal antibody (x axis), mouse anti-DR4 and anti-DR5 two monoclonal antibody (y axis). The cells were then incubated with FITC-conjugated rabbit anti-goat IgG and PE-conjugated rabbit anti-mouse IgG secondary antibody and analyzed by flow cytometry. (**B**) RFP expression profiles were determined in normal cells (CCD-11Lu) and carcinoma cells (Hela, MCF-7, ZR-75-30) infected with Ads. Cells were infected by Ads at an MOI of 5, and the RFP fluorescent signal was detected at various times post-infection by fluorescence microcopy, and morphological changes of recombinant Ad-infected cells were observed. A0, rAd5-RFP; A1, rAd5-zTRAIL-RFP; A2, rAd5pz-zTRAIL-RFP; A3, rAd5-zTRAIL-RFP-Δ24E1a; A4, rAd5pz-zTRAIL-RFP-Δ24E1a.

Our results thus far showed that TRAIL could induce tumor cell apoptosis effectively, and this effect was dependent on the virus dose. Therefore, in order to enhance the anti-tumor effects of the modified Ad, we constructed a CRAd with a tumor-specific 24-bp-deleted E1 gene under control of the survivin promoter, and rAd5-zTRAIL-RFP-SΔ24E1a was generated as a control (Figure 1C). Before analyzing the infection and replication of the modified oncolytic Ad vectors, the expression of TRAIL receptors (death receptors, DR4 and DR5; decoy receptors, DcR1 and DcR2) were detected by the flow cytometry assay in several cancer cells. Results showed that DR4/DR5 and DcR1/DcR2 were significantly more abundant on cancer cells than those on normal cells, and levels of DR4 and DR5 were higher than those of DcR1/2 (Figure 3A). To confirm the fluorescent signal of pIX-zippers-TRAIL proteins displayed on the recombinant Ads, CCD-11Lu cells were infected at 5 MOI, and the RFP activity was monitored by fluorescence microscopy 24 h and 48 h post-infection (Figure 3B). The results showed that the virus with modified pIX retained its fluorescent activity, and RFP protein expression levels were consistent with those of rAd5-RFP, rAd5-zTRAIL-RFP and rAd5-zTRAIL-RFP-SΔ24E1a. The infected CCD-11Lu cells did not vary greatly in shape, indicating that the CRAds had no toxic effects in normal cells. We also analyzed the effects of rAd5-zTRAIL-RFP-SΔ24E1a and rAd5pz-zTRAIL-RFP-SΔ24E1a on cell growth by fluorescence microscopy, and the RFP signals increased over time. Of note, RFP expression was dramatically increased in tumor cells infected by replication-competent Ads with survivin promoter, as compared to that in tumor cells infected by replication-deficient Ads. Moreover, compared with rAd5-zTRAIL-RFP-SΔ24E1a, infection with rAd5pz-zTRAIL-RFP-SΔ24E1a was enhanced efficiently and could induce apparent cytopathic effects in cancer cells. These results showed that the modified adenovirus vector was able to enhance expression of exogenous gene in different cancer cell lines, which with different expression levels of CAR and integrins receptors of Ad5 [32].

**Figure 4:**
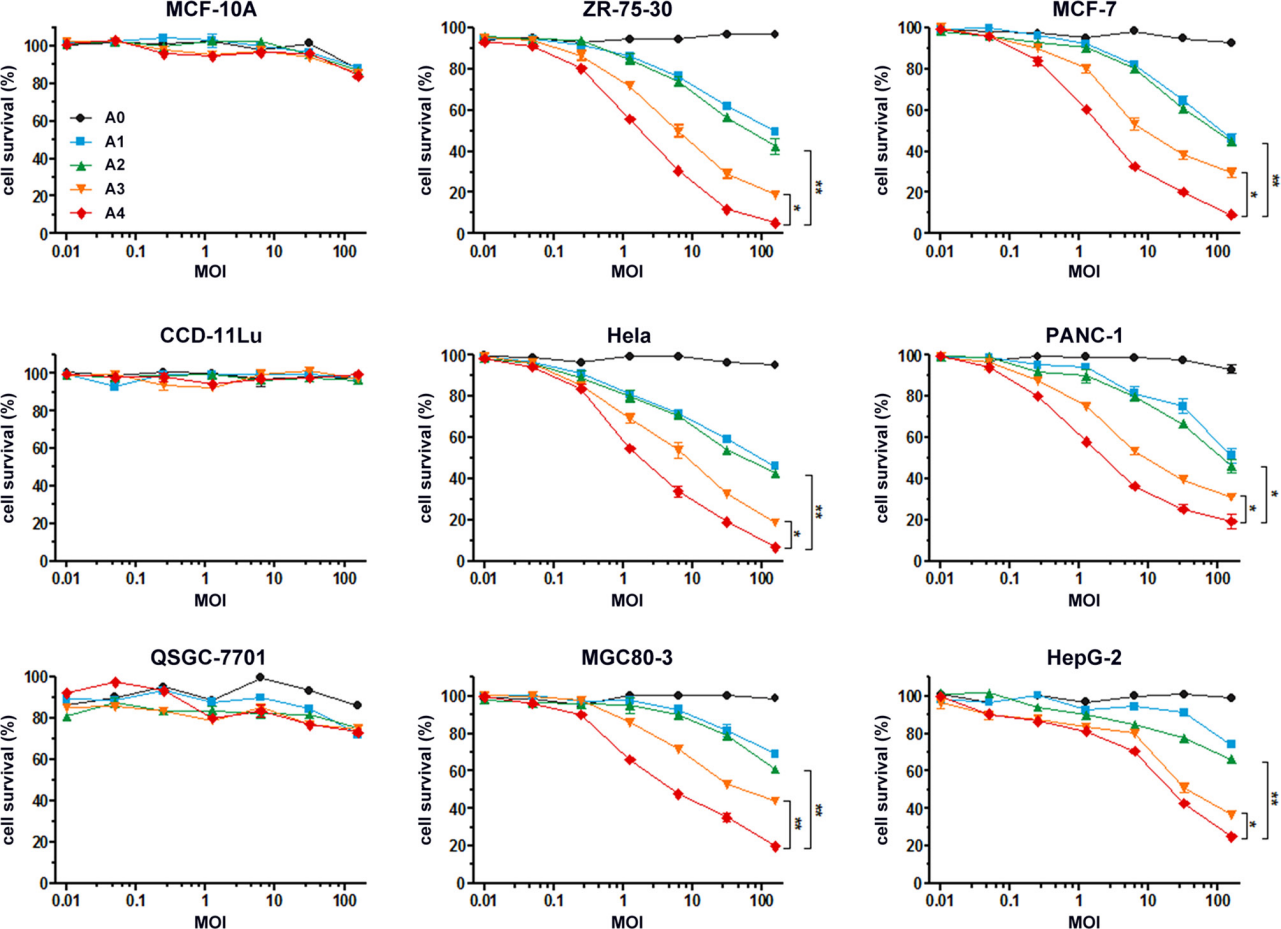
*In vitro* anti-tumor effects of recombinant Ad vectors Normal cells and tumor cells were infected with rAd5-RFP, rAd5-zTRAIL-RFP, rAd5pz-zTRAIL-RFP, rAd5-zTRAIL-RFP-SΔ24E1a or rAd5pz-zTRAIL-RFP-SΔ24E1a at different MOI (0.01, 0.05, 0.25, 1.25, 6.25, 32.25, 156.25) for 72 h. Cell survival rates were measured as described in Materials and Methods. All data represent the mean ± standard error of measurement (SEM) of three experiments. *P < 0.05; **P < 0.01.

To evaluate the bioactivity of recombinant Ads *in vitro*, we first analyzed their potential toxic effects in normal cells (i.e., MCF-10A, CCD-11Lu and QSGC-7701 cells) and found no cytotoxic activity (Figure 4). By contrast, incubation of recombinant Ads at different MOIs with different tumor cells revealed their ability to inhibit growth of cells, with rAd5pz-zTRAIL-RFP-SΔ24E1a exerting the greatest inhibitory effects. The results also showed an approximately 1.5-fold higher bioactivity of rAd5pz-zTRAIL-RFP-SΔ24E1a compared with the non-pIX-zipper CRAd. Of note, there had no significant difference between rAd5-zTRAIL-RFP and rAd5pz-zTRAIL-RFP in cytotoxic activity after a long time infection. These experiments showed that the modification of pIX with TRAIL and combined with a replication-competent Ad vector resulted in a stronger capacity to inhibit the growth of various types of cancer cells.

### Modified replication-competent Ad can target tumor tissues by IV injection *in vivo*

**Figure 5:**
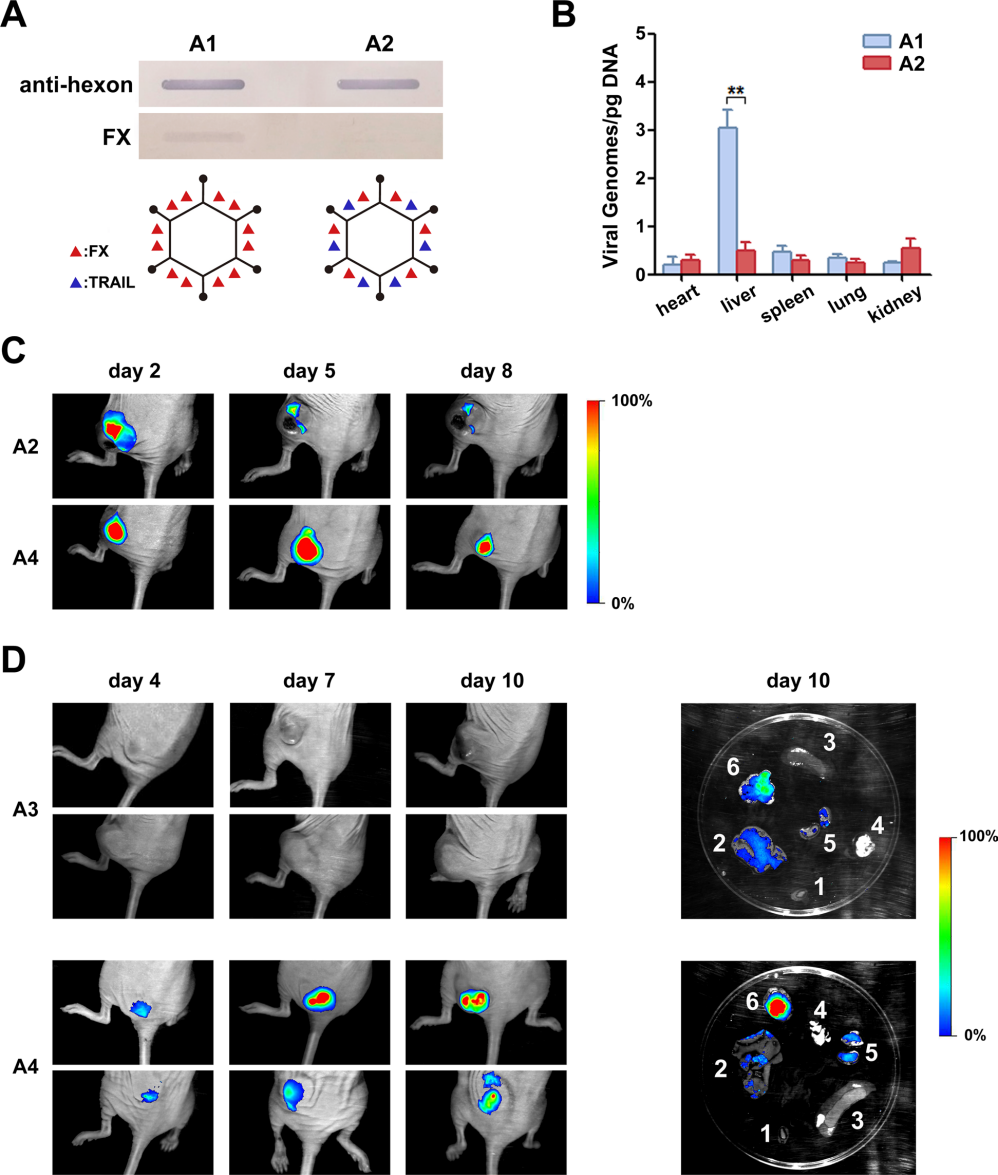
Analysis of FX binding with recombinant Ads and bioluminescent imaging of mice infected with RFP-expressing Ad containing a capsid-chained TRAIL protein (**A**) Slot blot assay and schematic diagram of FX binding with recombinant Ads. Viruses bound to a NC membrane were incubated with FX or with a polyclonal anti-Ad capsid antibody. The linkage of TRAIL with virus particles ablated the FX binding. (**B**) Mice were injected IV with 5 × 10^10^ PFU of rAd5-zTRAIL-RFP (**A1**) or rAd5pz-zTRAIL-RFP (**A2**). After 48 h, organs were isolated, and the virus distribution was detected by RT-PCR analysis. (**C**) Mice bearing ZR-75-30 breast tumors were injected IT with 1 × 10^9^ PFU of rAd5pz-zTRAIL-RFP (A2) and rAd5pz-zTRAIL-RFP-SΔ24E1a (**A4**). Images were captured over an 8-day period. (**D**) Athymic nude mice were implanted right flank with subcutaneous xenografts of ZR-75-30 cells. After tumor formation, mice were IV injected with 5 × 10^10^ PFU of rAd5-zTRAIL-RFP-SΔ24E1a (**A3**) or rAd5pz-zTRAIL-RFP-SΔ24E1a (A4). Images of representative mice from both groups are shown. On day 11, mice were sacrificed, and RFP expression levels in tumor tissues and organs were analyzed by bioluminescent imaging (right). 1, heart; 2, liver; 3, spleen; 4, lung; 5, kidney; 6, tumor tissue.

We further examined the binding of FX with the Ad capsid of rAd5-zTRAIL-RFP and rAd5pz-zTRAIL-RFP by slot blot assay, and little binding was detected between capsid-modified Ad5 and FX (Figure 5A). We inferred that pIX-TRAIL reduced the binding of FX with hypervariable regions of hexon due to the localization of pIXs between the hexons. To further evaluate whether the capsid-modified rAd could decrease liver tropism and transduction, nude mice were injected IV in the tail with rAds (rAd5-zTRAIL-RFP and rAd5pz-zTRAIL-RFP). After 48 h, biodistribution of rAds in each relevant organ was subjected to viral genome content analysis. Viral genome copies of rAd5pz-zTRAIL-RFP in the liver were significantly lower than those of rAd5-zTRAIL-RFP (Figure 5B). These results suggested that the modification of pIX in the Ad5 vector attenuated the liver tropism and intrahepatic transduction after IV administration.

In order to determine whether rAd5pz-zTRAIL-RFP-SΔ24E1a could replicate at a higher rate in tumor tissues, athymic nude mice were implanted with subcutaneous xenografts of the ZR-75-30 cell line. After 3 weeks, tumor nodules were injected intratumorally (IT) with rAd5pz-zTRAIL-RFP and rAd5pz-zTRAIL-RFP-SΔ24E1a and subsequently subjected to imaging analysis. RFP bioluminescence of rAds could be detected as early as day 2 after the injection. As shown in Figure 5C, bioluminescence of rAd5pz-zTRAIL-RFP-SΔ24E1a peaked on day 5 and began to decrease on day 8, while that of rAd5pz-zTRAIL-RFP was very weak two days later. The bioluminescent imaging experiment was repeated three times (each time *n* = 3) and the results had similar trend in the time (we just showed part of animal results).

To further verify the higher replication of rAd5pz-zTRAIL-RFP-SΔ24E1a in cancer cells, we next checked its ability to target tumor tissues *in vivo*. After the tail vein injection of CRAds, RFP bioluminescence of rAd5pz-zTRAIL-RFP-SΔ24E1a could be detected as early as day 4, and it increased over time in tumor tissues (ZR-75-30 xenografts) in the flanks of BALB/c nu/nu mice (Figure 5D, repeated three times, each time *n* = 3). By contrast, we did not detect fluorescent signals in tumor tissues of mice injected with rAd5-zTRAIL-RFP-SΔ24E1a. To substantiate that rAd5pz-zTRAIL-RFP-SΔ24E1a could target tumor tissues *in vivo* by IV injection, normal organs and tumor tissues were excised to detect RFP expression of CRAds on day 10 after the injection. A strong RFP fluorescent signal covering the whole tumor tissue in the rAd5pz-zTRAIL-RFP-SΔ24E1a group was observed, while the fluorescence was not observed in organs except for a weak signal the kidney and liver. By contrast, rAd5-zTRAIL-RFP-SΔ24E1a displayed a weak fluorescence in tumor and liver tissues. Thus, this strategy equipped CRAds with the capability of *in vivo* cancer selectivity and sustained replication by IV injection.

### Anti-tumor activity of Ads in a ZR-75-30 xenograft tumor model *in vivo*

**Figure 6:**
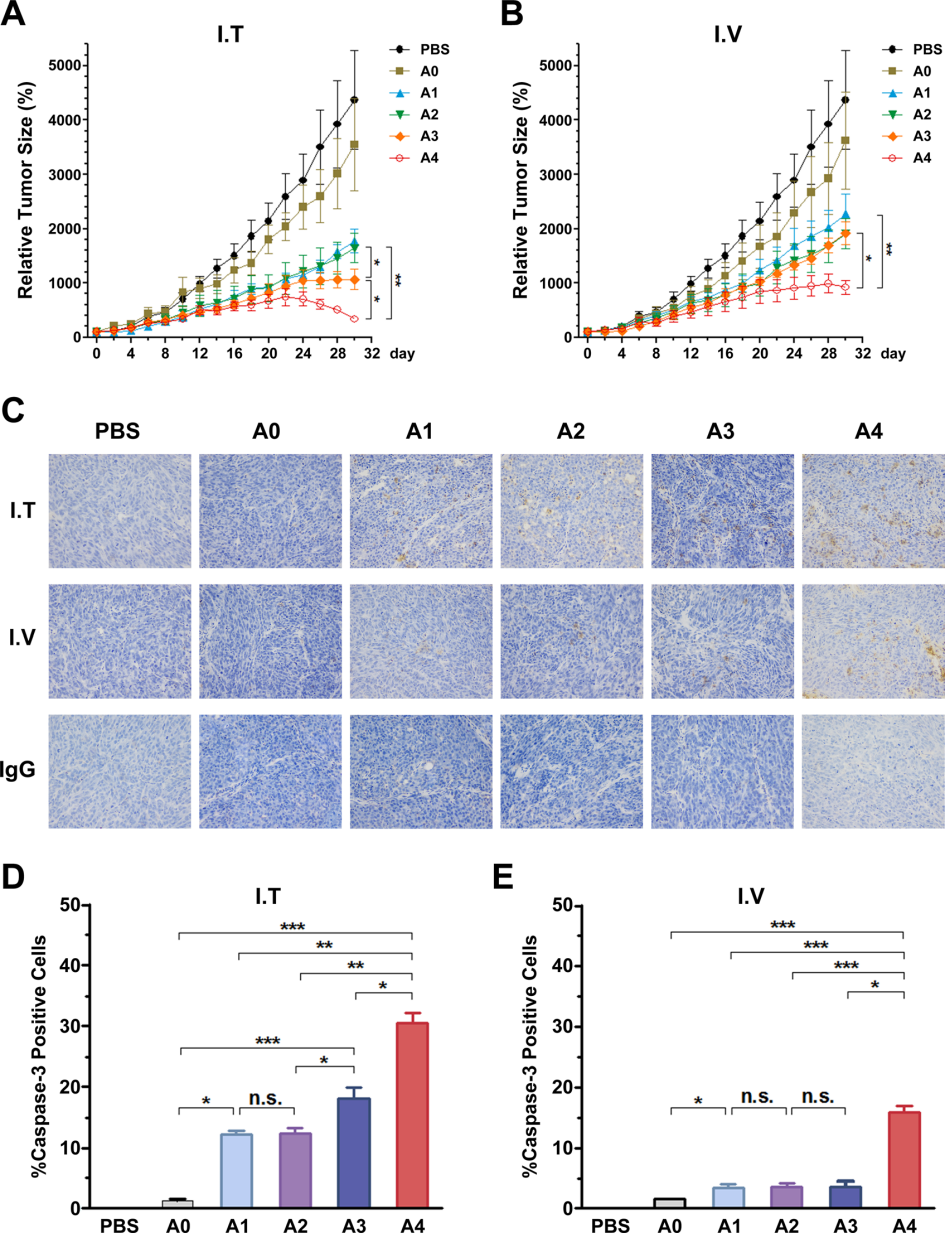
Anti-tumoral activity of Ads using different injection routes *in vivo*, and the detection of caspase-3 activation in tumor tissues treated with recombinant Ads by two routes in ZR-75-30 tumor-bearing mice (**A**) Anti-tumoral activity over time in a xenograft tumor model with local treatment. Mouse models received three daily IT injections of 1 × 10^8^ PFU of the corresponding Ad beginning on day 1. (**B**) Anti-tumoral activity over time in a xenograft tumor model with systemic treatment. Mouse models received three daily IV injections of 1 × 10^8^ PFU of the corresponding Ad beginning on day 1. *n* = 5 tumors per treatment group. Data points are means ± SEM of tumor volumes. Individual tumor sizes on day 30 with bars indicating the mean are shown on the right. (**C**) Detection of caspase-3 activation in tumor tissues treated with Ads by two different methods for 15 days. IgG control was reacted just without the caspase-3 antibody (this is only I.V group, and I.T group results were not showed because of the limited space). (**D**) and (**E**) Detection of caspase-3 activation in tumor tissues treated with Ads by two different methods for 15 days. Percentages of cells positive for active caspase-3 fragments assessed by analyzing three microscopic fields at 200 magnification (mean ± SEM). n.s., nonsignificant. A0, rAd5-RFP; A1, rAd5-zTRAIL-RFP; A2, rAd5pz-zTRAIL-RFP; A3, rAd5-zTRAIL-RFP-SΔ24E1a; A4, rAd5pz-zTRAIL-RFP-SΔ24E1a; *P < 0.05, **P < 0.01, ***P < 0.001.

To evaluate the anti-tumor potential of the vectors *in vivo*, we treated tumor-bearing mice with rAds via IT or IV injections. The growth kinetics of the tumors treated by the IT route are shown in Figure 6A. Compared with PBS and rAd5-RFP groups, other groups infected with rAds showed suppression of tumor growth. Especially in the rAd5pz-zTRAIL-RFP-SΔ24E1a infected group, tumor volume was significantly reduced at the later stage of treatment. By contrast, the rAd5-zTRAIL-RFP and rAd5pz-zTRAIL-RFP infected tumors grew slowly, with no significant difference between these two rAds. In mice injected via the IV route (Figure 6B), tumors of the rAd5pz-zTRAIL-RFP-SΔ24E1a infected groups decreased most significantly in size, compared with the PBS control and other recombinant Ad groups. Growth rates of the rAd5-zTRAIL-RFP, rAd5pz-zTRAIL-RFP and rAd5-zTRAIL-RFP-SΔ24E1a treated tumors were also suppressed, compared with rAd5-RFP infected groups; however, no significant difference in growth rates of the tumors in these groups was observed. The tumor size after treatment also showed the same results (Figure S1A).

To further support these results, we performed immunohistochemical (IHC) analyses for caspase-3 activation in tumor tissues of BALB/c nu/nu mice after treatment with rAds by injection via two different routes for 2 weeks. We first analyzed the IT injected group, and almost no caspase-3 activation was found in tumor tissues treated with PBS or rAd5-RFP. By contrast, tumor tissues of mice treated with rAd5pz-zTRAIL-RFP-SΔ24E1a revealed higher numbers of activated caspase-3-positive tumor cells, compared with other rAds treated animals (Figure 6C). In line with this observation, tumor tissues from mice treated IV with rAd5pz-zTRAIL-RFP-SΔ24E1a also showed higher levels of activated caspase-3, compared to tumor tissues from mice treated with other viruses (Figure 6C). To quantify the IHC results, cells positive for caspase-3 activation were counted. In contrast to rAd5-zTRAIL-RFP and rAd5pz-zTRAIL-RFP in the IT treatment group, treatment with CRAds resulted in a strong and significant (*P* < 0.05) increase of caspase-3 activation (rAd5-zTRAIL-RFP-SΔ24E1a, 18.12 ± 1.85%; rAd5pz-zTRAIL-RFP-SΔ24E1a, 30.47 ± 1.65%; Figure 6D). Compared to tumor tissues treated with replication-defective Ad vectors, treatment with rAd5-zTRAIL-RFP-SΔ24E1a via the IV route did not result in a significant increase of caspase-3 activation. In contrast, rAd5pz-zTRAIL-RFP-SΔ24E1a could induce a significant increase of caspase-3 activation (15.8 ± 1.2%; Figure 6E).

In experiments to assess targeting efficiency *in vivo*, we detected fluorescence signals of rAds in the liver and kidney. Therefore, safety of rAds by IV injection should be evaluated in these two organs. Fifteen days after gene transfer (Figure S1B), histological sections of the hepatic cord of mice treated with PBS and rAds revealed cells which were arranged orderly in a radiating pattern around the vein with no visible hepatotoxicity or hepatic necrosis. Meanwhile, histological sections of the kidney showed cortical renal cells arranged neatly and closely, with no morphological changes of the medulla renal cells.

**Figure 7:**
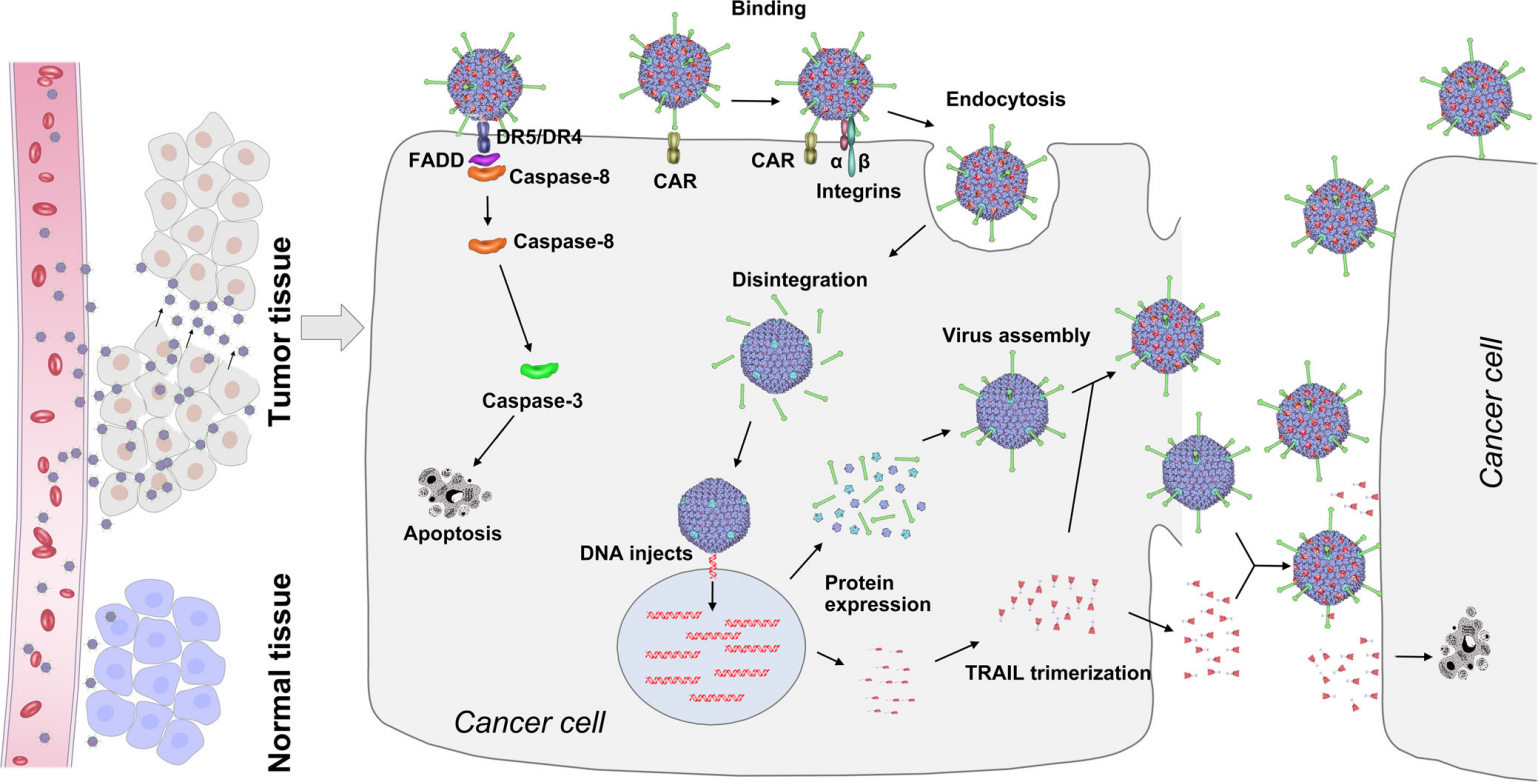
Schematic representation of mechanism of action of capsid-modified recombinant oncolytic Ads for cancer treatment Capsid-modified oncolytic recombinant Ads enter the bloodstream and are transported to the cancer site efficiently. Lifet: Due to the presence of TRAIL on the surface of viral particles, the recombinant Ads quickly infect and kill tumor cells but not normal cells. Right: TRAIL on the surface of modified oncolytic recombinant Ad particles can induce apoptosis in cancer cells. Massive replication occurs, and the viral progeny are released, leading to the cytolysis of cancer cells. Furthermore, TRAIL is secreted out of the cell and diffuses outward to other cancer cells. Thus, the new viruses with TRAIL can rapidly kill surrounding tumor cells.

Finally, we obtained a capsid-modified CRAd5 that could successfully target tumor tissues and exert prominent anti-tumor effects via the IV route. Its mechanisms are illustrated in Figure 7. The capsid-modified CRAd5 specifically recognizes and enters cancer cells. When capsid-modified CRAds infect cancer cells, the viral surface TRAIL induces programmed cell death by binding to its death receptors. The CRAd5 enters cancer cells and compels them to produce more viruses and abundant amounts of TRAIL, which can function in two ways. Isolated TRAIL proteins can recognize and induce apoptosis of cancer cells, while TRAIL proteins connected with pIX can aid capsid-modified CRAd5 in finding other tumors to destroy. In our strategy, TRAIL confers the tumor-targeting ability to CRAd5, and the capsid-modified CRAd in turn improves the stability of TRAIL and enhances its anti-tumor activity.

## DISCUSSION

Many limitations of oncolytic Ad gene therapy have been shown in preclinical trials, including targeting defects and low anti-tumor effects when administered IV, which would be the ideal delivery route for cancer therapy. Genetic modification of components of the Ad capsid, such as fiber, pIX or hexon, is an attractive strategy to alter the Ad tropism and obtain targeted viral vectors [9, 33–35]. pIX is an important target for modifying tropism, and its C terminus was found to effectively present large targeting polypeptides without affecting the stability of the virus [2, 36, 37].

In the current study, TRAIL was used to modify Ad pIX in order to increase the anti-tumor targeting, security and efficacy of the vector. Previous studies showed that a stable trimeric domain (such as leucine zipper) could serve as a fusion partner for improving the stability and cytotoxicity of the TRAIL protein [23]. Interestingly, three C termini of pIX gather in a structural unit, which is important for connecting the potential tumor-targeting TRAIL protein with the Ad particle. In this study, to protect the bioactivity of TRAIL in the induction of tumor cell apoptosis and to stabilize the protein structure, two complementary strands of a leucine zipper-like pair were used to effectively couple pIX and TRAIL. Meanwhile, the four-helix bundle of pIX (C-terminal region) could anchor the TRAIL proteins in the valley between two hexons from the outside of the Ad according to the crystal structure [30].

Recent studies have demonstrated the importance of coagulation factor X (FX) in adenovirus serotype 5-mediated liver transduction *in vivo* [11, 12]. Therefore, the pIX-modified Ad5 vector may have enhanced tumor targeting by mediating TRAIL binding to specific receptor moieties on the cancer cell surface, and liver enrichment of the virus may be reduced due to steric hindrance of the hexon–FX binding during IV administration. Conclusions from *in vitro* (Figure 5A) and *in vivo* (Figure 5B) data confirmed this speculation. Besides, slot blot results showed that modified TRAIL had no influence on the binding between rAd5 and anti-hexon, hence, capsid-modified oncolytic rAd5 might be unlikely to facilitate evasion of pre-existing anti Ad5 immunity. However, due to the hexon-specific neutralizing antibodies not the main role when adenovirus infect human naturally [38, 39], the virion in this manner could be suitable for low-infected and uninfected population. Additionally, other interactions in the blood influencing viral tropism, such as complement proteins [40] and natural antibodies [41] also should be considered and need to be further investigated.

To obtain more effective Ad vectors for cancer therapy, we should enhance their tumor targeting ability in the body as well as improve their anti-tumor effects. In the current study, we adopted the Gene-Viro-Therapy (CTGVT) approach of cancer targeting, as it has demonstrated much higher anti-tumor effects [42, 43]. Many reports have shown that TRAIL could induce the selective death in a wide range of human cancer cells [44–46]. Therefore, researchers have used TRAIL as a foreign gene for cancer treatment in CTGVT research [47–49]. Although all of those treatments were effective when tested *in vivo* in nude mouse xenografts by IT injection [42, 43, 50], these studies did not carry out the animal experiments with IV injection of the vector, and such data are necessary for clinical evaluation. Furthermore, CTGVT research tended to show effective treatment of hepatic carcinoma but without discussing the problem of liver enrichment if the vector needs to be used to target other tissues [33]. In this work, we demonstrated that the modified pIX with the leucine zipper efficiently assembled into the virus particles, and the TRAIL protein could successfully be linked to the viral particle of the capsid-modified oncolytic recombinant Ad. In this case, the advantage of rAd5pz-zTRAIL-RFP-SΔ24E1a is to enable the oncolytic Ad to target tumors, thereby facilitating the specific and efficient killing of cancer cells. Moreover, TRAIL can replicate together with oncolytic viral vectors in tumor tissues, thereby greatly enhancing the ability to induce cancer cell death. Overall, rAd5pz-zTRAIL-RFP-SΔ24E1a can effectively migrate to the cancerous site after IV injection, and then persistently express foreign genes and generate progeny virus. Thus, TRAIL is not merely an anti-cancer drug as Kaczorowski et al. study [51], but also functions as a tumor targeting molecule when coupled with Ad. Encouragingly, TRAIL present on the surface of viral particles reduces the binding of FX to the hypervariable regions of hexons and decreases liver transduction.

Taken together, the capsid-modified oncolytic recombinant Ad showed potent anti-cancer effects by IV injection. This finding indicates that rAd5pz-zTRAIL-RFP-SΔ24E1a may be combined with other chemotherapy drugs, particularly apoptosis inducers or anti-apoptosis inhibitors, which will potentially improve its efficacy and safety further in clinical trials. Therefore, we believe that rAd5pz-zTRAIL-RFP-SΔ24E1a may be a promising candidate for the treatment of various cancers in clinical practice. Our strategy offers a basis and new research direction for the application of Ad as a carrier in cancer gene therapy, particularly in IV treatments for different types of cancer.

## MATERIALS AND METHODS

### Cell lines and cell culture

The following human cell lines were purchased from American Type Culture Collection (ATCC): HEK293, human embryonic kidney cells; CCD-11Lu, human lung fibroblasts; MCF-10A, human breast epithelial cells; ZR-75-30 and MCF-7, human breast adenocarcinoma cells; PANC-1, human pancreatic carcinoma cells; Hela, human cervical carcinoma cells; MGC80-3, human gastric adenocarcinoma cells; HepG-2, human hepatoma cells. QSGC-7701, human Hepatocytes, was purchased from the Cell Bank of the Type Culture Collection of the Chinese Academy of Sciences (Shanghai, China). All the cell cultures were accorded with guidelines of ATCC, and all cell lines used in this study were authenticated using short tandem repeat (STR) profiling less than 6 months ago when this project was initiated, and the cells have not been in culture for more than 2 months.

### Virus construction and production

Sequence analysis showed one *Sma* I site upstream and one downstream of the protein pIX termination codon (5370, 8013). Accordingly, we carried out the following construction steps. First, Ad gene fragments with pIX sequences were transferred into the pET-20b vector using the *Xba* I and *Bst*1107 restriction sites. Second, the original gene was replaced with the synthesized *Sma* I-linker (G4S) 3-zipper (R-R34)-*Sma* I fragment using the restriction enzyme *Sma* I. The insertion was confirmed by PCR using the primers 5′-AAGAATATATAAGGTGGGGGTC-3′ (forward) and 5′-GATGTTACGACAACGTCCA AC-3′ (reverse). Finally, the mutated fragment was inserted into the viral DNA vector pAdMax at the *Xba* I and *Bst*1107 I sites. The resulting construct was the Ad vector carrying the linker (G4S) 3-zipper (R-R34) in the C terminal end of pIX. Using synthetic gene assembly technology, the zipper (E-E34)-linker (G4S) 3 was added to the N-terminus of TRAIL. We then constructed three shuttle plasmids pDC316-RFP, zTRAIL-RFP and zTRAIL-RFP-SΔ24E1a. The three plasmids were each co-transfected with the viral vector Ad5-pIX-zipper (+ / −) into 293 cells for homologous recombination and packaging of five recombinant viruses: Ad5-RFP, Ad5-zTRAIL-RFP, Ad5pz-zTRAIL-RFP, Ad5-zTRAIL-RFP-SΔ24E1a and Ad5pz-zTRAIL-RFP-SΔ24E1a. The genome sizes: CMV-RFP, 1211 bp; CMV-zTRAIL, 1515 bp; SUR-Δ24E1a, 4007 bp. All virions were purified using CsCl equilibrium density gradient centrifugation, comparative analysis of Ad particle size was used twice CsCl gradient centrifugation as described [52]. The Physicochemical titers were determined by the 260-nm absorbance method [53], and PFU titers were determined by the TCID50 method [54].

### Immunoelectron microscopy

Viruses were fixed with 2% paraformaldehyde/PBS at room temperature for 10 min and adhered to 400-mesh copper grids supported with carbon-coated Formvar film (EMS). After washing with 1% BSA/PBS twice for 5 min each, grids were probed with a 1:200 diluted anti-TRAIL antibody (Abcam, Cambridge, UK) and incubated at 4°C overnight. After 2 cycles of 1%BSA/PBS washes, grids were incubated with 1:40 diluted 5 nm gold-conjugated goat anti-rabbit antibodies (Boster, Wuhan, China) at room temperature for 30 min. After fixing with 1% glutaraldehyde/PBS for 20 min, grids were subjected to negative staining in 2% phosphotungstic acid for 12 s and examined under a transmission electron microscope at a high resolution imaging facility.

### ELISA

Ninety-six-well plates coated overnight at 4°C with 100 μl/well of a mouse monoclonal antibody (Clone: sc-51588, Santa Cruz Biotechnology, CA, USA) raised against sTRAIL (100 ng/ml) (freeze-dried powder, Sino Biological Inc., Beijing, China) in PBS were blocked with 2% BSA/PBS-T (20 mM PBS containing 0.05% Tween-20). Serial dilutions of TRAIL protein and rAd vectors were then added in excess and incubated at 37°C. The plates were then incubated with a 1/2000 dilution of rabbit polyclonal antibody (Santa Cruz Biotechnology, CA, US) to TRAIL at 37°C. The plates were then incubated with a 1/5000 dilution of a peroxidase-conjugated affinity-purified goat anti-rabbit secondary antibody (Proteintech Group, Rosemont, IL, USA). After every step the plates were washed three times with PBS-T and eventually developed with tetramethylbenzidine (TMB), stopped with 2 M H_2_SO_4_, and analyzed at double wavelengths 450-630 nm with an EL × 800 Universal Microplate reader (Bio-Rad Laboratories).

### Flow cytometry analysis of TRAIL's receptors

Cultured cells were washed and harvested with PBS, resuspended in PBS containing 1% BSA for 30 min at room temperature, and then incubated with anti-DR4 and anti-DR5 (both of them are mouse monoclonal antibodies, Clone: sc-32255 and sc-51588, Santa Cruz Biotechnology, CA, USA), and anti-DcR1/2 (goat polyclonal antibody, Santa Cruz Biotechnology, CA, USA) antibodies for 1 h on ice. Subsequently, the cells were washed and incubated with mouse anti-goat IgG-FITC and goat anti-mouse IgG1-PE (Santa Cruz Biotechnology, CA, USA) for an additional 30 min. After washing with 1% BSA/PBS, the cells were analyzed by flow cytometry with the MoFlo XDP cell sorter (Beckman Coulter). The same secondary antibodies alone served as the negative control for each cell line.

### Fluorescence assay

Ad-transduced ZR-75-30 cells (MOI = 10, 30, 100 or 300) and non-transduced control cells were plated in 96-well plates at a density of 1 × 10^4^ cells/well in 100 μl RPMI1640 culture medium for 6 h. Fluorescent immunostaining was performed on cells double-stained with FITC-labeled Annexin V and propidium iodide (PI) following the manufacturer's instructions of the Annexin V-FITC Apoptosis Detection Kit (Beckman Coulter).

### Flow cytometry analysis of apoptosis

Early apoptotic cells were detected using the Annexin V-PI Apoptosis Kit (Beckman Coulter, Krefeld, Germany). Cells were treated with Ads at the MOI of 100, harvested after 2, 4, 6 or 8 h and washed with PBS. The cells were then resuspended in binding buffer, stained with Annexin V (0.6 mg/ml) and PI (5 mg/ml) for 15 min in the dark at room temperature and analyzed by two-color flow cytometry.

### Cell viability assay

Ad-induced cell death was assessed using the MTT assay. Cells were infected with recombinant Ads at various concentrations (MOI = 0.01, 0.05, 0.25, 1.25, 6.25, 32.25, 156.25), and the cell viability was measured after 72 h. The survival ratio (%) was calculated according to the following formula: [(experimental group absorbance - background absorbance)/(control group absorbance - background absorbance)] ×100%.

### Slot blot analysis

Virus particles (1 × 10^11^ vp) were exposed to a nitrocellulose (NC) membrane, which was pre-incubated with TBS, using a slot blot chamber. The NC membrane was washed five times with TBST (0.2% Tween 20) and then incubated with blocking reagent (1% skimmed milk in TBS) for 30 min at room temperature. The membrane was then incubated with either purified human FX (8 μg/ml) (Haematologic Technologies Inc, Essex Junction, VT, USA) or an adenovirus type 5 hexon polyclonal antibody (1:1000) (Thermo Scientific, MA, USA) for 1 h at room temperature. The membranes were washed five times with TBST between all incubations and incubated with 1% TBS blocking reagent for 30 min at room temperature prior to exposure to the next antibody. The FX-treated samples were incubated with anti-FX monoclonal mouse antibodies (Clone: AHX-5050, Haematologic Technologies Inc, Essex Junction, VT, USA) followed by anti-mouse goat-HRP antibodies (DAKO, Carpinteria, CA, USA). Samples incubated with anti-hexon antibodies were subsequently incubated with an anti-rabbit goat-HRP antibody (DAKO, Carpinteria, CA, USA). Following a final wash cycle, NC membranes were developed with DAB (Sigma, Beijing, China).

### Xenograft tumor model in nude mice

Female BALB/c nude mice at 4–5 weeks old were obtained from Vital River Laboratories (VRL, Beijing, China) and quarantined for a week before tumor implantation. All procedures were performed according to institutional guidelines and conformed to the National Institutes of Health guidelines on the ethical use of animals. Freshly cultured ZR-75-30 cells (2 × 10^6^) were injected subcutaneously into the right flank of mice. When the tumors had grown to 100–200 mm^3^, the animals were randomized into twelve groups (8 mice/group). Each group was treated with either PBS or 1 × 10^8^ PFU of vectors IT or IV for three consecutive days. At the second week after the first injection, two tested mice were randomly selected and killed to harvest tumors for additional analyses as described below. The tumor size was measured using a vernier caliper every two days. The tumor volume (mm^3^) was calculated as (length × width^2^)/2. At the end of the experiment, all of the tested animals were sacrificed. Differences in tumor growth were evaluated for statistical significance.

### *In vivo* fluorescent imaging

Mice with breast cancer were IT injected with 1 × 10^9^ virus PFU/mouse, or were IV injected with 5 × 10^10^ virus PFU/mouse. Over the next few days, mice were subjected to whole-body bioluminescent quantification (Kodak FX PRO), and were killed after observations. The tissue peeled for biodistribution analysis of recombinant Ads in each relevant organ.

### Viral genome content of tissues

DNA was extracted from tissues using the QIAamp DNA mini kit (QIAGEN, Beijing, China) following the manufacturer's instructions. DNA was quantified using Nanodrop spectophotometer (ThermoScientific) and 100 ng of DNA containing viral genomes were quantified using SyBR green real-time polymerase chain reaction (PCR; 7900HT Sequence Detection System; Applied Biosystems) using a concentration of 0.2 μM hexon primers.

### IHC analysis

The tumor sections were incubated with goat anti-cleaved caspase 3 antibodies (diluted 1:150) (Clone: C92-605, Cell Signaling, Boston, MA, USA). After incubation with an anti-rabbit secondary antibody, signals were detected with DAB (Sigma, Beijing, China) and enhanced with an avidin-biotin reaction ABC kit (Vector Laboratories, Burlingame, CA, USA). And the primary antibodies were omitted in the IgG control. The slides were then counterstained with hematoxylin.

### Statistical analysis

Values were expressed as the mean ± standard deviation (SD), and statistical analysis of the results was carried out using one-way analysis of the variance (ANOVA) followed by Duncan's new multiple range method or Newman-Keuls test. *P*-values < 0.05 were considered significant.

## SUPPLEMENTARY MATERIALS AND METHODS



## Abbreviations

Ad: adenovirus
rAd: recombinant adenovirus
CRAd: conditionally replicating adenoviruses
PFU: plaque forming unit
MOI: multiplicity of infection
IV: intravenous
IT: intratumoral
ANOVA: analysis of variance

## References

[1] Kaufmann JK, Nettelbeck DM (2012). Virus chimeras for gene therapy vaccination and oncolysis: adenoviruses and beyond. Trends in molecular medicine.

[2] Russell SJ, Peng KW, Bell JC (2012). Oncolytic virotherapy. Nat Biotechnol.

[3] Avila MA, Berasain C, Sangro B, Prieto J (2006). New therapies for hepatocellular carcinoma. Oncogene.

[4] Kimura J, Ono HA, Kosaka T, Nagashima Y, Hirai S, Ohno S, Aoki K, Julia D, Yamamoto M, Kunisaki C, Endo I (2013). Conditionally replicative adenoviral vectors for imaging the effect of chemotherapy on pancreatic cancer cells. Cancer Sci.

[5] Pesonen S, Kangasniemi L, Hemminki A (2011). Oncolytic adenoviruses for the treatment of human cancer: focus on translational and clinical data. Mol Pharm.

[6] Ugai H, Wang M, Le LP, Matthews DA, Yamamoto M, Curiel DT (2010). *In vitro* dynamic visualization analysis of fluorescently labeled minor capsid protein IX and core protein V by simultaneous detection. J Mol Biol.

[7] Wang SB, Tan Y, Lei W, Wang YG, Zhou XM, Jia XY, Zhang KJ, Chu L, Liu XY, Qian WB (2012). Complete eradication of xenograft hepatoma by oncolytic adenovirus ZD55 harboring TRAIL-IETD-Smac gene with broad antitumor effect. Hum Gene Ther.

[8] Short JJ, Rivera AA, Wu H, Walter MR, Yamamoto M, Mathis JM, Curiel DT (2010). Substitution of adenovirus serotype 3 hexon onto a serotype 5 oncolytic adenovirus reduces factor X binding decreases liver tropism and improves antitumor efficacy. Mol Cancer Ther.

[9] Coughlan L, Alba R, Parker AL, Bradshaw AC, McNeish IA, Nicklin SA, Baker AH (2010). Tropism-modification strategies for targeted gene delivery using adenoviral vectors. Viruses.

[10] Poulin KL, Lanthier RM, Smith AC, Christou C, Risco Quiroz M, Powell KL, O‘Meara RW, Kothary R, Lorimer IA, Parks RJ (2010). Retargeting of adenovirus vectors through genetic fusion of a single-chain or single-domain antibody to capsid protein IX. J Virol.

[11] Alba R, Bradshaw AC, Parker AL, Bhella D, Waddington SN, Nicklin SA, van Rooijen N, Custers J, Goudsmit J, Barouch DH, McVey JH, Baker AH (2009). Identification of coagulation factor (F)X binding sites on the adenovirus serotype 5 hexon: effect of mutagenesis on FX interactions and gene transfer. Blood.

[12] Waddington SN, McVey JH, Bhella D, Parker AL, Barker K, Atoda H, Pink R, Buckley SM, Greig JA, Denby L, Custers J, Morita T, Francischetti IM (2008). Adenovirus serotype 5 hexon mediates liver gene transfer. Cell.

[13] Parker AL, Waddington SN, Nicol CG, Shayakhmetov DM, Buckley SM, Denby L, Kemball-Cook G, Ni S, Lieber A, McVey JH, Nicklin SA, Baker AH (2006). Multiple vitamin K-dependent coagulation zymogens promote adenovirus-mediated gene delivery to hepatocytes. Blood.

[14] Fabry CM, Rosa-Calatrava M, Moriscot C, Ruigrok RW, Boulanger P, Schoehn G (2009). The C-terminal domains of adenovirus serotype 5 protein IX assemble into an antiparallel structure on the facets of the capsid. J Virol.

[15] Parks RJ (2005). Adenovirus protein IX: a new look at an old protein. Mol Ther.

[16] Mahalingam D, Oldenhuis CN, Szegezdi E, Giles FJ, de Vries EG, de Jong S, Nawrocki ST (2011). Targeting TRAIL towards the clinic. Curr Drug Targets.

[17] Abe K, Kurakin A, Mohseni-Maybodi M, Kay B, Khosravi-Far R (2000). The complexity of TNF-related apoptosis-inducing ligand. Ann N Y Acad Sci.

[18] Holland PM (2013). Targeting Apo2L/TRAIL receptors by soluble Apo2L/TRAIL. Cancer Lett.

[19] Martinez-Lostao L, Marzo I, Anel A, Naval J (2012). Targeting the Apo2L/TRAIL system for the therapy of autoimmune diseases and cancer. Biochem Pharmacol.

[20] Almasan A, Ashkenazi A (2003). Apo2L/TRAIL: apoptosis signaling biology and potential for cancer therapy. Cytokine Growth Factor Rev.

[21] Gonzalvez F, Ashkenazi A (2010). New insights into apoptosis signaling by Apo2L/TRAIL. Oncogene.

[22] Pan LQ, Wang HB, Xie ZM, Li ZH, Tang XJ, Xu YC, Zhang C, Naranmandura H, Chen SQ (2013). Novel conjugation of tumor-necrosis-factor-related apoptosis-inducing ligand (TRAIL) with monomethyl auristatin E for efficient antitumor drug delivery. Adv Mater.

[23] Pan LQ, Xie ZM, Tang XJ, Wu M, Wang FR, Naranmandura H, Chen SQ (2013). Engineering and refolding of a novel trimeric fusion protein TRAIL-collagen XVIII NC1. Appl Microbiol Biotechnol.

[24] Siegemund M, Pollak N, Seifert O, Wahl K, Hanak K, Vogel A, Nussler AK, Gottsch D, Munkel S, Bantel H, Kontermann RE, Pfizenmaier K (2012). Superior antitumoral activity of dimerized targeted single-chain TRAIL fusion proteins under retention of tumor selectivity. Cell Death Dise.

[25] Spitzer D, McDunn JE, Plambeck-Suess S, Goedegebuure PS, Hotchkiss RS, Hawkins WG (2010). A genetically encoded multifunctional TRAIL trimer facilitates cell-specific targeting and tumor cell killing. Mol Cancer Ther.

[26] Garas MN, Tillib SV, Zubkova OV, Rogozhin VN, Ivanova TI, Vasilev LA, Logunov DY, Shmarov MM, Tutykhina IL, Esmagambetov IB, Gribova IY, Bandelyuk AS, Naroditsky BS (2014). Construction of a pIX-modified Adenovirus Vector Able to Effectively Bind to Nanoantibodies for Targeting. Acta naturae.

[27] Suzuki K, Fueyo J, Krasnykh V, Reynolds PN, Curiel DT, Alemany R (2001). A conditionally replicative adenovirus with enhanced infectivity shows improved oncolytic potency. Clin Cancer Res.

[28] Cody JJ, Rivera AA, Lyons GR, Yang SW, Wang M, Sarver DB, Wang D, Selander KS, Kuo HC, Meleth S, Feng X, Siegal GP, Douglas JT (2010). Arming a replicating adenovirus with osteoprotegerin reduces the tumor burden in a murine model of osteolytic bone metastases of breast cancer. Cancer Gene Ther.

[29] Suzuki K, Alemany R, Yamamoto M, Curiel DT (2002). The presence of the adenovirus E3 region improves the oncolytic potency of conditionally replicative adenoviruses. Clin Cancer Res.

[30] Liu H, Jin L, Koh SB, Atanasov I, Schein S, Wu L, Zhou ZH (2010). Atomic structure of human adenovirus by cryo-EM reveals interactions among protein networks. Science.

[31] Glasgow JN, Mikheeva G, Krasnykh V, Curiel DT (2009). A strategy for adenovirus vector targeting with a secreted single chain antibody. PloS one.

[32] Zhan Y, Yu B, Wang Z, Zhang Y, Zhang HH, Wu H, Feng X, Geng RS, Kong W, Yu XH (2014). A fiber-modified adenovirus co-expressing HSV-TK Coli NTR enhances antitumor activities in breast cancer cells. Int J Clin Exp Pathol.

[33] Campos SK, Parrott MB, Barry MA (2004). Avidin-based targeting and purification of a protein IX-modified metabolically biotinylated adenoviral vector. Mol Ther.

[34] Kurachi S, Koizumi N, Sakurai F, Kawabata K, Sakurai H, Nakagawa S, Hayakawa T, Mizuguchi H (2007). Characterization of capsid-modified adenovirus vectors containing heterologous peptides in the fiber knob protein IX or hexon. Gene Ther.

[35] Kurachi S, Tashiro K, Sakurai F, Sakurai H, Kawabata K, Yayama K, Okamoto H, Nakagawa S, Mizuguchi H (2007). Fiber-modified adenovirus vectors containing the TAT peptide derived from HIV-1 in the fiber knob have efficient gene transfer activity. Gene Ther.

[36] Vaha-Koskela MJ, Heikkila JE, Hinkkanen AE (2007). Oncolytic viruses in cancer therapy. Cancer Lett.

[37] Vellinga J, de Vrij J, Myhre S, Uil T, Martineau P, Lindholm L, Hoeben RC (2007). Efficient incorporation of a functional hyper-stable single-chain antibody fragment protein-IX fusion in the adenovirus capsid. Gene Ther.

[38] Cheng C, Gall JG, Nason M, King CR, Koup RA, Roederer M, McElrath MJ, Morgan CA, Churchyard G, Baden LR, Duerr AC, Keefer MC, Graham BS (2010). Differential specificity and immunogenicity of adenovirus type 5 neutralizing antibodies elicited by natural infection or immunization. J Virol.

[39] Yu B, Dong J, Wang C, Zhan Y, Zhang H, Wu J, Kong W, Yu X (2013). Characteristics of neutralizing antibodies to adenovirus capsid proteins in human and animal sera. Virology.

[40] Carlisle RC, Di Y, Cerny AM, Sonnen AF, Sim RB, Green NK, Subr V, Ulbrich K, Gilbert RJ, Fisher KD, Finberg RW, Seymour LW (2009). Human erythrocytes bind and inactivate type 5 adenovirus by presenting Coxsackie virus-adenovirus receptor and complement receptor 1. Blood.

[41] Xu Z, Qiu Q, Tian J, Smith JS, Conenello GM, Morita T, Byrnes AP (2013). Coagulation factor X shields adenovirus type 5 from attack by natural antibodies and complement. Nat Med.

[42] Liu XY, Gu JF (2006). Targeting gene-virotherapy of cancer. Cell Res.

[43] Liu XY, Li HG, Zhang KJ, Gu JF (2012). Strategy of Cancer Targeting Gene-Viro-Therapy (CTGVT) a trend in both cancer gene therapy and cancer virotherapy. Curr Pharm Biotechnol.

[44] Chae SY, Kim TH, Park K, Jin CH, Son S, Lee S, Youn YS, Kim K, Jo DG, Kwon IC, Chen X, Lee KC (2010). Improved antitumor activity and tumor targeting of NH(2)-terminal-specific PEGylated tumor necrosis factor-related apoptosis-inducing ligand. Mol Cancer Ther.

[45] Wahl K, Siegemund M, Lehner F, Vondran F, Nussler A, Langer F, Krech T, Kontermann R, Manns MP, Schulze-Osthoff K, Pfizenmaier K, Bantel H (2013). Increased apoptosis induction in hepatocellular carcinoma by a novel tumor-targeted TRAIL fusion protein combined with bortezomib. Hepatology.

[46] Stuckey DW, Shah K (2013). TRAIL on trial: preclinical advances in cancer therapy. Trends Mol Med.

[47] Wei RC, Cao X, Gui JH, Zhou XM, Zhong D, Yan QL, Huang WD, Qian QJ, Zhao FL, Liu XY (2011). Augmenting the antitumor effect of TRAIL by SOCS3 with double-regulated replicating oncolytic adenovirus in hepatocellular carcinoma. Hum Gene Ther.

[48] Cao X, Yang M, Wei RC, Zeng Y, Gu JF, Huang WD, Yang DQ, Li HL, Ding M, Wei N, Zhang KJ, Xu B, Liu XR (2011). Cancer targeting Gene-Viro-Therapy of liver carcinoma by dual-regulated oncolytic adenovirus armed with TRAIL gene. Gene Ther.

[49] Liu X, Cao X, Wei R, Cai Y, Li H, Gui J, Zhong D, Liu XY, Huang K (2012). Gene-viro-therapy targeting liver cancer by a dual-regulated oncolytic adenoviral vector harboring IL-24 and TRAIL. Cancer gene Ther.

[50] Huang F, Ma B, Wang Y, Xiao R, Kong Y, Zhou X, Xia D (2014). Targeting gene-virus-mediated manganese superoxide dismutase effectively suppresses tumor growth in hepatocellular carcinoma *in vitro* and *in vivo*. Cancer Biother Radi.

[51] Kaczorowski A, Hammer K, Liu L, Villhauer S, Nwaeburu C, Fan P, Zhao Z, Gladkich J, Gross W, Nettelbeck DM, Herr I (2016). Delivery of improved oncolytic adenoviruses by mesenchymal stromal cells for elimination of tumorigenic pancreatic cancer cells. Oncotarget.

[52] Ostapchuk P, Almond M, Hearing P (2011). Characterization of Empty adenovirus particles assembled in the absence of a functional adenovirus IVa2 protein. J Virol.

[53] Sweeney JA, Hennessey JP (2002). Evaluation of accuracy and precision of adenovirus absorptivity at 260 nm under conditions of complete DNA disruption. Virology.

[54] Yu B, Zhang Y, Zhan Y, Zha X, Wu Y, Zhang X, Dong Q, Kong W, Yu X (2011). Co-expression of herpes simplex virus thymidine kinase and Escherichia coli nitroreductase by an hTERT-driven adenovirus vector in breast cancer cells results in additive anti-tumor effects. Oncol Rep.

