# Supplementary material for: A novel capsid-modified oncolytic recombinant adenovirus type 5 for tumor-targeting gene therapy by intravenous route
Source: Oncotarget. 2016 Jun 15;7(30):47287–301. doi: 10.18632/oncotarget.10075 (PMC5216942; doi:10.18632/oncotarget.10075)
Supplement: Supplementary file 1 [file oncotarget-07-47287-s001.pdf]

A novel capsid-modified oncolytic recombinant adenovirus type 5 for tumor-targeting gene therapy by intravenous route

Supplementary Materials

MATERIALS AND METHODS

IHC analysis

All slide-mounted tissue sections of livers and kidneys were stained with hematoxylin-eosin (H&E) and examined microscopically to determine pathological changes.

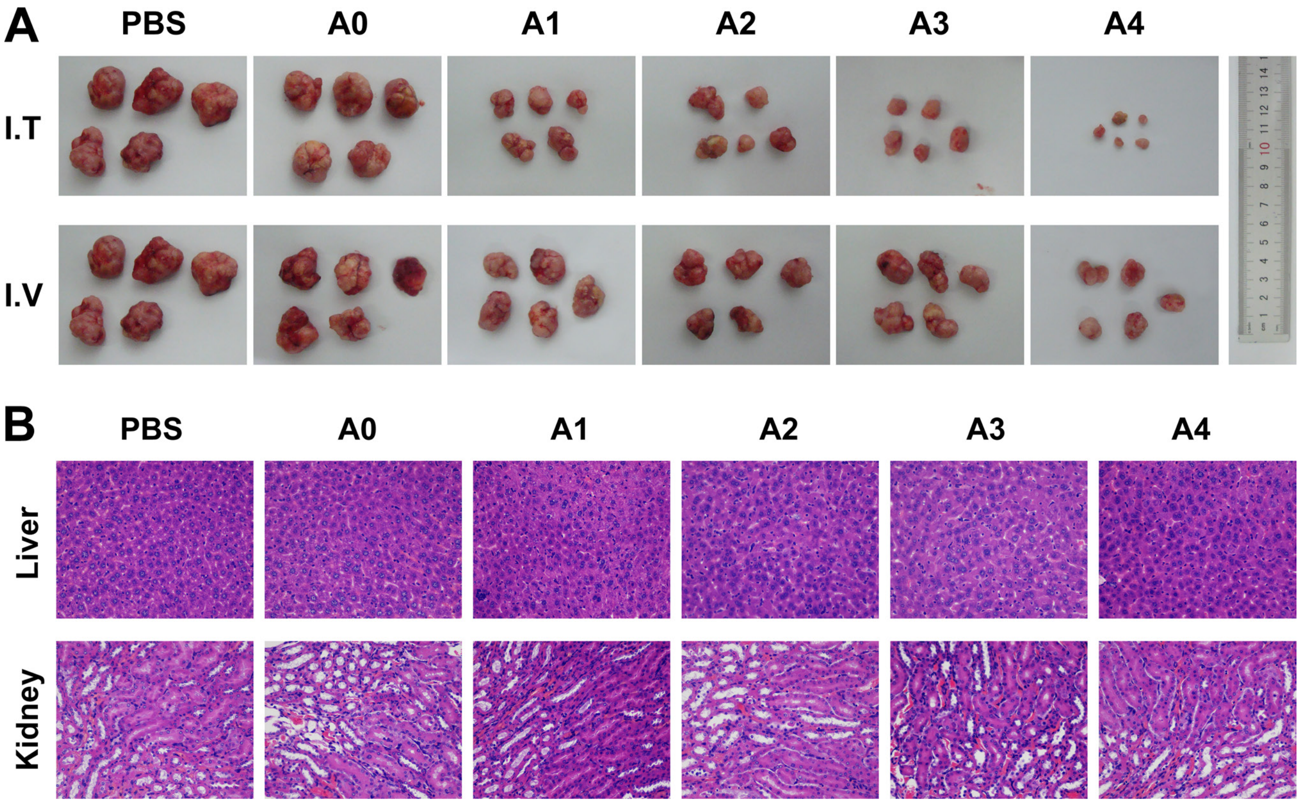

Supplementary Figure S1: The tumor size and histological analysis of livers and kidneys after treatment. (**A**) Tumor tissues measured on day 31 after treatment. (**B**) Mice were injected IV with PBS and Ads. Fifteen days later, livers and kidneys were isolated, and histological analyses were performed using H&E staining.
